# Henoch-Schönlein purpura with acute pancreatitis: analysis of 13 cases

**DOI:** 10.1186/s12887-018-1142-7

**Published:** 2018-05-11

**Authors:** Qin Zhang, Qi Guo, Ming Gui, Zhenhua Ren, Bo Hu, Ling Lu, Fang Deng

**Affiliations:** 1 Department of Pediatrics, First Affiliated Hospital of Anhui Medical University, 19th Floor of Medicine and Medical Tech Building, 218 Jixi Road, Hefei, 230022 Anhui China; 2 Department of Anatomy, School of Basic Medicine, Anhui Medical University, Hefei, 230032 Anhui China

**Keywords:** Henoch-Schönlein purpura, Acute pancreatitis, Complication, Children

## Abstract

**Background:**

Henoch-Schönlein purpura is a common small vessel vasculitis in children. Acute pancreatitis rarely presents as a complication of Henoch-Schönlein purpura and has not been well characterized.

**Methods:**

We retrospectively reviewed 13 cases of Henoch-Schönlein purpura with acute pancreatitis among 3212 patients who attended our hospital between January 2003 and June 2016 and analyzed their clinical characteristics, laboratory findings, imaging findings, treatment and overall prognosis.

**Results:**

All patients had abdominal manifestations, including significant abdominal pain (13/13), vomiting (9/13), abdominal distension (3/13) and melena (6/13). Serum amylase level significantly increased in all patients, and urine amylase was increased in 7 cases (7/10). However, increased urine lapse was only noted in 2 cases (2/5), and diffuse swelling of the pancreas was seen in 2 cases (2/13) by abdominal ultrasonography. Although all patients had typical skin purpura (13/13), 5 patients (5/13) with acute pancreatitis initially experienced acute abdominal pain in clinical onset of Henoch-Schönlein purpura. Glucocorticoid therapy was effective in alleviating abdominal symptoms of Henoch-Schönlein purpura patients with acute pancreatitis. All patients were in good general condition without any abdominal complications 6–12 months after discharge.

**Conclusions:**

Acute pancreatitis is rarely observed in Henoch-Schönlein purpura children and has no specific clinical features that differentiate it from abdominal manifestations of Henoch-Schönlein purpura. Therefore, in Henoch-Schönlein purpura patients with severe abdominal pain, serum amylase levels should be assessed to confirm the diagnosis of acute pancreatitis. Early diagnose of Henoch-Schönlein purpura with acute pancreatitis and treatment timely was very important for good clinical outcomes.

## Background

Henoch-Schönlein purpura (HSP), a systemic small-vessel vasculitis syndrome, typically occurs in children and causes purpuric skin lesions accompanied by various presentations, including gastrointestinal symptoms, arthritis, and nephritis [1]. The severity of symptoms and the order of their appearance differ among patients. Gastrointestinal manifestations occur in two-thirds of patients and vary from mild abdominal pain to colicky features [2].

Acute pancreatitis (AP) has been reported by only a limited number of authors, with different clinical features during the course of HSP. Most cases of HSP pancreatitis are mild in clinical characteristics but can evolve into hemorrhage, necrosis and pseudocyst [3, 4]. As a complication, AP can be diagnosed on the basis of a high level of serum amylase in HSP patients with abdominal pain. In cases with epigastric tenderness, routine serum and urine amylase evaluations may reveal pancreatic involvement in HSP [4]. We herein retrospectively reviewed 13 cases of HSP patients with AP among 3212 HSP patients and analyzed symptoms and signs, laboratory findings, imaging findings, treatment and their prognosis.

## Methods

All the clinical data used in this study were obtained from the paper and electronic medical records (EMRs) of our hospital. The study protocol was approved by the Research Ethics Commission of the First Affiliated Hospital of Anhui Medical University.

### Patients

Children with a final diagnosis of HSP presenting to the Department of Pediatrics, First Affiliated Hospital of Anhui Medical University were prospectively enrolled between January 2003 and June 2016. All patients conformed to the standard established by the European League Against Rheumatism/Paediatric Rheumatology International Trials Organization/Paediatric Rheumatology European Society [5]. All patients had typical skin purpura over the buttocks and lower extremities and exhibited 1 or more of the typical manifestations of the syndrome, including abdominal symptoms, hematuria, or arthritis. The diagnosis of AP was made in patients with two of the following three manifestations: characteristic upper abdominal pain, elevated levels of pancreatic enzymes, or imaging findings suggesting AP [6].

### Data collection

We reviewed the age, length of hospital stay, and initial manifestation of HSP patients with AP. The abdominal manifestations of HSP among patients with AP, including abdominal pain, nausea, vomiting, upper gastrointestinal bleeding manifested with tarry stool passage, hematochezia and diarrhea, were analyzed. Laboratory studies including serum and urine amylase levels, hemogram, blood calcium, fecal occult blood, and imaging reports were recorded. The treatment process and outcome were also reviewed.

## Results

### Clinical features

Three thousand two hundred twelve HSP children, including 1966 males and 1249 females (mean age 6.7 ± 2.41 years old, absolute range 1.4–15 years), were enrolled in the study. Among the 3212 HSP patients, only 13 patients (0.4%), including 9 males and 4 females (mean age 10.58 ± 2.63 years old, absolute range 6–14 years), had AP as a complication of HSP. Eight cases with AP had typical rash for HSP in the onset (8/13), and the time interval between skin purpura and identification of pancreatic damage was from 2 to 30 days (the average was 9.25 ± 9.65 days). Five cases with AP initially experienced acute abdominal pain (5/13), and skin purpura appeared 4–15 days after disease onset (the average was 7.40 ± 4.83 days). Details regarding age, duration of hospital stay, and initial manifestation are presented in Table 1.

**Table 1:** The age, hospital stay time, and initial manifestation in 13 HSP children

case number	Hospital stay (d)	Initial manifestation	Time interval between skin purpura and finding pancreatitis (d)	Time between pancreatitis and skin purpura (d)
1	45	AAp	–	3
2	28	AAp	–	4
3	12	AAp	–	9
4	3	SP	2	–
5	5	SP	5	–
6	15	SP	30	–
7	11	SP	15	–
8	33	AAp	–	15
9	44	SP	3	–
10	24	AAp	–	6
11	25	SP	12	–
12	17	SP	5	–
13	9	SP	2	–

*M* Male, *F* Female, *SP* skin purpura, *AAp* acute abdominal pain

In all HSP patients with AP, we encountered two uncommon cases. They (Case No. 1 and No. 2) initially experienced acute abdominal pain and underwent operations due to the suspicion of acute appendicitis or cholecystitis (Table 1). Their purpuric skin rashes appeared later than the presentation of abdominal pain. HSP was diagnosed after the appearance of a typical rash.

All HSP patients with AP exhibited a palpable purpura rash, and the purpura typically appeared on the buttocks and the extensor surfaces of the arms and legs. However, any area of the body may be involved. As shown in Table 2, significant abdominal pain and tenderness were common abdominal manifestations in HSP patients with AP. The periumbilical area was most common location of abdominal tenderness. Six cases (6/13) exhibited upper gastrointestinal tract bleeding presenting with tarry stool passage (Table 2). In addition, arthritis involvement was noted in 7 patients (7/13), and nephritis presenting with proteinuria occurred in 10 patients (10/13).

**Table 2:** Clinical features of abdominal symptoms among 13 children with Henoch-Schönlein purpura and acute pancreatitis

Clinical features		No. of patients	Percent (%)
Colicky abdominal pain		13	100
Abdominal tenderness		13	100
Abdominal tenderness location	periumbilical area	6	46
	upper abdomen	1	7.7
	whole abdomen	3	23
	No definite position	3	23
Abdominal distension		3	23
Abdominal wall tension		3	23
Nausea and vomiting		9	69
Upper gastrointestinal tract bleeding		6	46
Melena		6	46
Hematemesis		1	7.7
Diarrhea		1	7.7
Arthritis		7	54
nephritis		10	77

### Laboratory studies

Laboratory values are presented in Table 3. Of note, serum amylase was elevated in all patients with AP. Moreover, urine amylase levels increased in 7 cases (7/10), and serum lipase levels only increased in 2 cases (2/5). As shown in Table 3, all patients with AP had an abnormal hemogram. None of the patients had a bleeding tendency or coagulopathy. Other examinations, including imaging examination, electrocardiogram, myocardial enzymes and liver function, were performed in some HSP patients with AP. Their results are depicted in Table 4.

**Table 3:** The Laboratory values in 13 HSP children

case number	Serum amylase (< 110 IU/L)	Urine amylase (< 900 U/L)	Serum lipase (< 300 U/L)	White blood cell count (< 10 × 10^9^/L)	Neutrophil ratio (< 70%)	Blood calcium (> 2.03 mmol/L)	Occult blood (+)
1	132	–	–	22	78	1.6	2(+)
2	382	8687	845	17	78	2.3	1(+)
3	182	2600	–	16	67	2.6	(−)
4	365	80	–	21	88	–	(−)
5	222	775	–	10	60	2.4	–
6	546	4411	32	29	88	2.5	(−)
7	143	941	–	20	80	2.6	(−)
8	174	1210	–	15	79	1.9	2(+)
9	325	1330	258	22	83	2.0	3(+)
10	162	–	–	18	86	2.0	2(+)
11	193	3524	548	12	83	2.4	(+)
12	121	–	–	20	67	2.3	–
13	175	446	61	19	81	2.3	(+)

(+): positive; (−): Negative; −--: No test

**Table 4:** Additional examinations in 13 children with Henoch-Schönlein purpura and acute pancreatitis

case number	Abdominal radiograph	Abdominal ultrasonography	CT	ECG	Myocardial enzymes
1	BD	PE	–	AN	AN
2	N	N	–	N	N
3	–	N	–	N	N
4	–	PE	N	N	N
5	N	N	N	AN	AN
6	–	N	–	N	N
7	–	N	–	N	N
8	BD	PDS	–	N	N
9	–	PE	–	AN	AN
10	N	PE	–	N	N
11	N	N	N	N	N
12	–	PDS	–	N	N
13	–	N	PDS	N	N

*CT* Computerized tomography, *ECG* Electrocardiogram, *BD* Bowel dilatation, *PDS* Pancreatic diffuse swelling, *PE* Peritoneal effusion, *AN* Abnormality, *N* Normal; ---: No test

### Treatment

Methyl prednisolone pulse therapy was used for HSP patients presenting with AP. The patients were treated with methyl prednisolone (10–15 mg/kg/day) every other day 3 times during a treatment course, and one or two additional treatment courses were considered depending on the conditions of the patients. Moreover, for the treatment of AP, H2 receptor antagonists or proton pump inhibitors are used to inhibit the secretion of gastric acid. The anti-secretory agents (octreotide) and anti-inflammatory drugs (amoxicillin and clavulanate potassium) were used to prevent pancreatic enzyme secretion and anti-bacterial infection, respectively. According to the available guidelines for the treatment of AP, supportive care was pivotal. In addition, pain management should be mentioned when caring for a patient with AP.

After methyl prednisolone pulse therapy, the symptoms of abdominal pain were alleviated, and serum and urine amylase levels returned to normal. Then, prednisolone (1 mg/kg/day) was administered orally until urine test were normal and the symptoms had completely disappeared. The average duration of treatment of the HSP patients with methyl prednisolone and prednisolone was 18.56 ± 8.18 days, and the total course of treatment ranged from 3 to 45 days.

### Outcome and follow-up

After methyl prednisolone pulse therapy and the treatment of AP, purpuric skin rashes and gastrointestinal symptoms disappeared in all HSP children with AP. Serum amylase recovered to normal levels from 3 to 6 days after treatment, and urine amylase levels returned to normal in 3–13 days.

In the follow-up period of 6–12 months, no recurrence of AP or pancreatic cyst formation was observed in any of the HSP children. However, the recurrence of abdominal pain was noted in 4 children (1 episode in 3 patients and 2 episodes in 1 patient). The interval between the first episode and recurrence ranged from 12 to 44 days with a mean time of 19.3 days. All of these patients with recurrence were treated with corticosteroids. Three patients developed Henoch–Schönlein purpura nephritis (HSPN).

## Discussion

Henoch–Schönlein purpura (HSP) is a systemic vasculitis that is characterized by the deposition of IgA-containing complex and complement component 3 (C3) on arterioles, capillaries, and venules [7]. Clinical manifestations observed in HSP, including skin purpura, abdominal pain, arthritis, and glomerulonephritis, are typically attributed to widespread vasculitis in the small vessels, which causes an increase in vascular permeability and fragility [8].

HSP complicated by acute pancreatitis (AP) occurs rarely, and the exact cause is unknown. The pathophysiologic mechanism of AP is thought to be vasculitic involvement of the capillaries, small arteries and small veins within pancreatic tissues, which increases vascular permeability and leads to pancreatic edema [8–10]. Embolism in the small vessels of the pancreas causes pancreatic ischemia and hypoxia and the abnormal activation of digestive enzymes. These effects lead to inflammation, edema, vascular injury, and even cellular death [11].

AP in HSP was generally recorded as case reports in previous studies [10, 12–15]. Among all HSP complications, the incidence of AP is very low. In a study of 208 patients with HSP, Chen SY et al. report gastrointestinal manifestations and complications of HSP. Among these patients, only one case (0.48%) exhibited highly elevated serum amylase and lipase levels [2]. A recent study reviewed that 50 case reports documented the involvement of the pancreato-biliary system in HSP, and found that only 23 cases among of records in the US National Library of Medicine database from 1977 to 2015 had pancreatitis in HSP [15]. In our study, we identified only 13 children with AP among 3212 HSP patients (0.4%) during the past 14 years. Because serum amylase and lipase levels have not been routinely measured in all HSP cases, the incidence of AP may be higher than our data [16].

To the best of our knowledge, the reported 13 patients represent the biggest retrospective case series hitherto published. The complication of pancreatitis can occur as an initial manifestation of HSP. Frigui et al. analyzed pediatric HSP cases with pancreatitis, and identified AP as an initial manifestation in half of cases [4, 10, 17, 18]. In this study, 5 HSP children among 13 patients (38%) exhibited an onset of abdominal symptoms. The other 8 HSP cases with AP (62%) first presented with the characteristic rash. Elevated serum amylase levels are an early diagnostic finding for HSP pancreatitis [17]. Serum amylase and lipase levels might be more important and should be measured in patients who present with severe abdominal pain as the initial complaint. Ultrasonography or CT signs, such as swollen pancreas, edema and necrosis, are useful to confirm the diagnosis only if pancreatitis is severe. In our study, although serum and urine amylase levels increased significantly in all HSP patients with AP, only 2 cases (15%) exhibited diffuse swelling of the pancreas by ultrasonography.

Pancreatic involvement in HSP is mild in nature and self-limiting. According to Working Group IAP/APA Acute Pancreatitis Guidelines, oral feeding can be initiated as soon as the pain decreases and inflammatory markers are lowering [19]. Octreotide is a synthetic analogue of somatostatin that is effective for the treatment of AP via the inhibition of the release of exocrine secretions of the pancreas [20]. Steroid administration has been controversial in the treatment of AP over the past few decades. The evidence indicates that steroid administration worsened pancreatitis and should not be applied in AP [4]. However, the results of experimental animal models of AP demonstrated that steroids have an inhibitory effect on pancreatic secretion [21] and inhibit the development of AP partly via cytokine release [22]. Steroid therapy can be used in HSP patients with AP and is effective in alleviating symptoms of AP [4, 16, 18]. However, the previous study demonstrated that corticosteroid use was associated with an increased risk of gastrointestinal bleeding and perforation [23]. Therefore, HSP patients, especially children complicated with gastrointestinal bleeding, must be closely followed up during steroid treatment [4]. In our study, all the patients with AP were treated with methyl prednisolone and achieved a good outcome. No patient experienced a gastrointestinal complication during the course of steroid therapy.

## Conclusions

HSP patients with severe abdominal pain should be evaluated for pancreatitis. Serum amylase level is a useful tool for the early diagnosis of acute pancreatitis. Although good outcome can be achieved in most HSP patients, supportive treatment for HSP pancreatitis and maybe also steroid therapy might be performed to avoid unnecessary surgical interventions.

## Abbreviations

AP: Acute pancreatitis
CT: Computerized tomography
EMR: Electronic medical record
EULAR: European League Against Rheumatism
HSP: Henoch–Schönlein purpura
HSPN: Henoch–Schönlein purpura nephritis
MRI: Magnetic resonance imaging
PReS: Pediatric Rheumatology Society
US: Ultrasonography
